# Microglia in the crosstalk between peripheral and central nervous systems in Parkinson’s disease

**DOI:** 10.1186/s40035-025-00531-3

**Published:** 2025-12-11

**Authors:** Tianbai Li, Tao Qiu, Fei Jiang, Huaibin Cai, Weidong Le

**Affiliations:** 1 Key Laboratory of Liaoning Province for Research On the Pathogenic Mechanisms of Neurological Diseases, The First Affiliated Hospital of Dalian Medical University, Dalian, 116021 China; 2 Dalian Seventh People’s Hospital, Dalian, 116023 China; 3 Transgenic Section, Laboratory of Neurogenetics, National Institute on Aging, National Institutes of Health, Bethesda, MD 20892 USA; 4 Neurology Program, Sir Run Run Shaw Hospital, Zhejiang University School of Medicine, Hangzhou, 310016 China

**Keywords:** Microglia, Parkinson’s disease, Inflammation, α-Synuclein, Gut microbial metabolites

## Abstract

Parkinson’s disease (PD) is increasingly recognized as a multisystem disorder involving pathological α-synuclein (α-syn) accumulation and widespread neuroimmune dysregulation. Microglia, the resident immune cells in the central nervous system (CNS), are pivotal mediators of the bidirectional communication between the CNS and peripheral systems. In addition to sensing neuronal injury and α-syn pathology, microglia dynamically respond to peripheral immune signals, including circulating cytokines, immune cell infiltration, and microbial metabolites, through pattern recognition receptors such as Toll-like and NOD-like receptors. Furthermore, microglia regulate blood–brain barrier integrity, modulate peripheral immune cell recruitment, interact with meningeal lymphatic vessels, and contribute to the propagation of α-syn within the CNS and along the gut–brain axis. However, a comprehensive framework encompassing their diverse roles in peripheral–central immune crosstalk remains underdeveloped. This review synthesizes recent advances elucidating how microglia link the CNS to peripheral immune and metabolic signals in PD. We further highlight microglial contributions to α-syn propagation along the gut–brain axis and discuss how their functional states influence disease progression. A deeper understanding of microglial involvement in this complex neuroimmune interface may inform the development of effective and system-level therapeutic strategies for PD.

## Introduction

Parkinson’s disease (PD) is a progressive neurodegenerative disorder primarily characterized by the degeneration of dopaminergic (DAergic) neurons in the substantia nigra pars compacta (SNc), accompanied by a wide range of motor and non-motor symptoms [1, 2]. Pathological α-synuclein (α-syn) aggregates are not restricted to the central nervous system (CNS), but are also widely distributed in the peripheral nervous system (PNS), including the enteric nervous system (ENS), the vagus nerve, and sympathetic ganglia [3–5]. These findings support the concept that PD is a multisystem disorder involving both the CNS and the PNS.

In parallel with the α-syn propagation hypothesis, increasing evidence highlights a prominent role for immune dysregulation in PD [6]. The CNS is no longer viewed as an immune-privileged organ but rather having bidirectional communications with the peripheral immune system [7]. Peripheral immune system dysregulation including gut dysbiosis, systemic inflammation, and altered immune cell profiles, is closely linked to PD pathogenesis and progression [8].

Microglia are the CNS-resident immune cells that function as dynamic sensors and effectors of the neuroimmune environment [9, 10]. Beyond their traditional role in maintaining homeostasis and responding to local neuronal injury, microglia are increasingly recognized to mediate the bidirectional communications between the CNS and peripheral compartments [11]. Genome-wide association studies have revealed many genes specifically or strongly expressed in microglia as risk factors for neurodegenerative diseases [11, 12]. In PD, peripheral immune signals, such as circulating cytokines, microbial metabolites, and infiltrating immune cells, can access the CNS particularly when the blood–brain barrier (BBB) integrity is compromised [13]. Microglia are equipped with receptors that can sense these signals and translate them into neuroinflammatory responses, which in turn exacerbate α-syn pathology and impair neuronal survival [10]. Moreover, microglia actively modulate BBB integrity, regulate immune cell infiltration, interact with border-associated macrophages (BAMs) at the meninges and choroid plexus, therefore participating in the propagation of α-syn pathology [7]. Furthermore, microglia contribute to the propagation of α-syn pathology both within the CNS and along the gut–brain axis, serving as a key interface linking peripheral immune alterations to central neurodegeneration in PD [14].

While microglial activation has been extensively investigated in PD, a comprehensive delineation of the multifaceted roles of microglia within the context of peripheral–CNS communication is lacking. Here, we conduct a narrative literature review based on a systematic search of the PubMed/Medline database, including peer-reviewed studies published from 2015 to 2025. Search terms included “Parkinson’s disease,” “microglia,” “inflammation,” “α-synuclein,” “meningeal-associated macrophages” and “gut microbiota”. This review summarizes microglial interactions with DAergic neurons, peripheral immune signals, and the meningeal lymphatic system, as well as their roles in α-syn propagation and the gut–brain axis. Furthermore, we highlight how microglia integrate multifactorial signals to adopt dynamic functional states that influence disease progression. Finally, we outline therapeutic strategies targeting the microglia–periphery interactions for disease modification in PD.

## Role of microglia in the peripheral–central immune crosstalk in PD

**Fig. 1:**
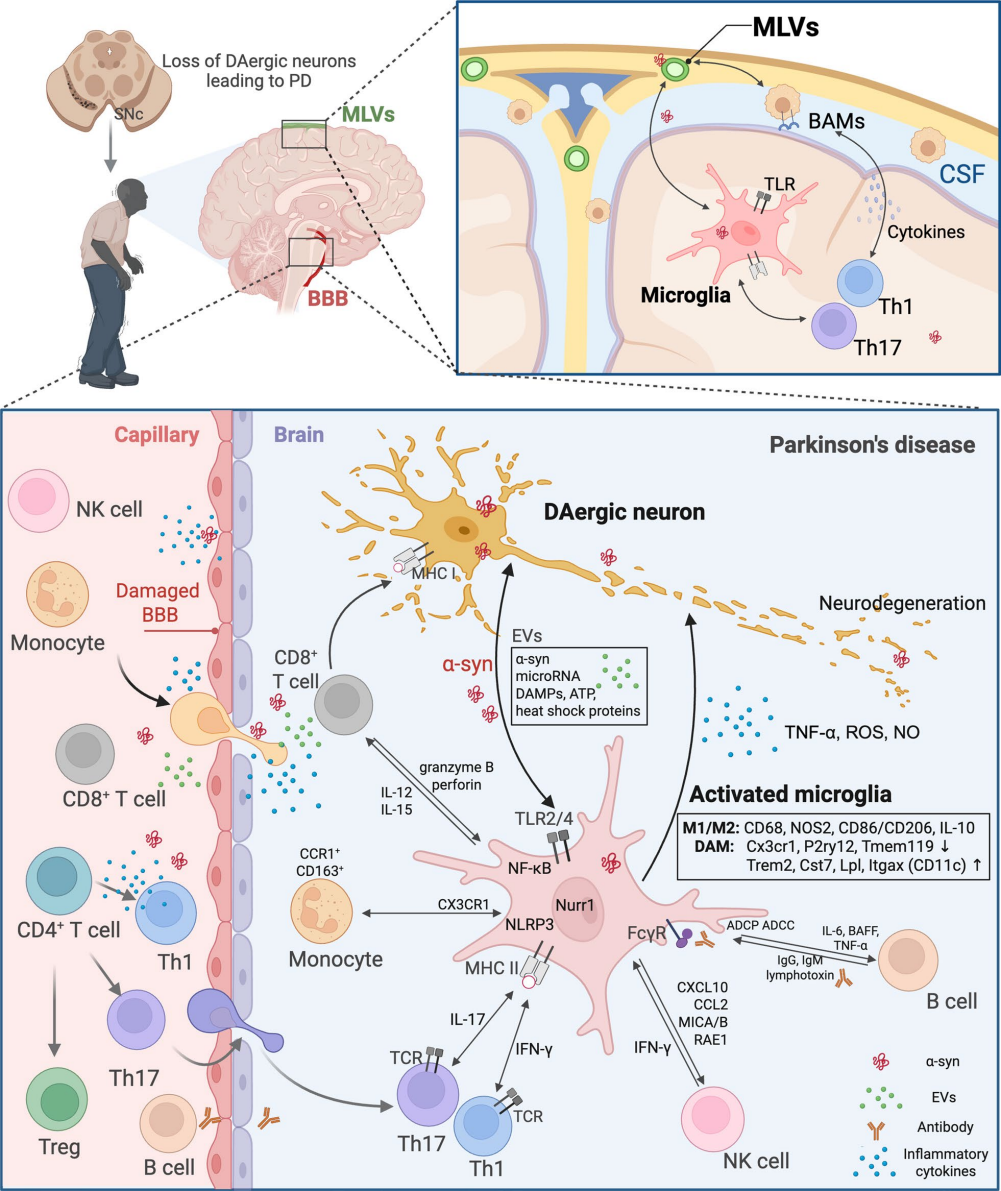
Roles of microglia in the crosstalk between central and peripheral immune systems. Under pathological conditions, peripheral immune cells, including CD4^+^ T cells, CD8^+^ T cells, Tregs, NK cells, monocytes and B cells, can infiltrate the CNS via compromised BBB or through meningeal and perivascular routes. These immune cells modulate the activation states, transcriptional profiles, and effector functions of microglia. In parallel, microglia regulate the peripheral immune tone by secreting cytokines and chemokines that influence immune cell recruitment and polarization. Border-associated macrophages, located at the meninges, perivascular spaces, and choroid plexus, serve as key antigen-presenting cells linking CNS-derived antigens (including pathological α-syn) to peripheral adaptive immune activation via the meningeal lymphatic vessels (MLVs). This dynamic neuroimmune crosstalk plays a critical role in driving PD pathogenesis and progression. Figure created with BioRender.com

Emerging clinical and preclinical evidence supports a dynamic and bidirectional interplay between the central and the peripheral immune systems in PD, in which microglia serve as  a critical intermediary coordinating this neuroimmune communication [7, 11]. Peripheral immune signals can modulate microglial activation through various mechanisms, including disruption of the BBB, recruitment of peripheral immune cells into the CNS, and interactions with BAMs (Fig. 1) [15]. In turn, microglia shape the peripheral immune tone by secreting cytokines, chemokines, and other soluble mediators, which may influence peripheral immune cell programming and trafficking [11]. Understanding the context and the temporal features of microglia-mediated central–peripheral crosstalk is essential for elucidating PD immunopathogenesis.

### Microglia–DAergic neuron interactions in PD

Microglia are increasingly recognized to be central to PD pathogenesis through early sensing of neuronal dysfunction and activation of neuroinflammation [10]. Evidence from postmortem brain tissues of PD patients demonstrates a consistent pattern of microglial activation in affected regions such as the SNc, characterized by morphological change, proliferation, and dysregulation of inflammatory markers [10, 16]. In vivo positron emission tomography (PET) imaging studies using 18-kDa translocator protein (TSPO) ligands like PK11195 further provided visualization of activated microglia and confirmed widespread neuroinflammatory responses in PD brains [17].

There is increasing evidence of bidirectional communication between microglia and DAergic neurons [18]. Under physiological conditions, neurons maintain the homeostatic, surveillant state of microglia through inhibitor signals such as chemokine (C-X3-C motif) ligand 1 (CX3CL1) and CD200 [18]. During the prodromal state of PD, early synaptic dysfunction and metabolic stress in DAergic neurons result in impairment of these regulatory cues. In response, microglia undergo phenotypic reprogramming shaped by the local neuronal environment, transitioning toward reactive states with distinct transcriptional and functional profiles [19]. These dynamic changes can influence the roles of microglia (neuroprotective or neurotoxic), depending on the contextual factors such as disease stage and regional cues.

In addition to functioning as sentinels of neuronal distress, microglia-mediated inflammation can exacerbate the DAergic neurodegeneration in PD [16]. In genetic and toxin-induced PD models, microglial activation precedes overt DAergic neurodegeneration [20, 21]. In addition, microglia-specific deletion of nuclear receptor related 1 (Nurr1), a PD-associated transcription factor with anti-inflammatory properties, exacerbates α-syn aggregation and DAergic neurodegeneration, underscoring the causal role of microglial dysregulation in disease progression [22, 23].

Microglia exert neurotoxic effects through both soluble factors and extracellular vesicles (EVs) [24]. EVs released by activated microglia, including exosomes and microvesicles, can carry inflammatory mediators, neurotoxic miRNAs, and pathogenic α-syn species [25]. These vesicles are taken up by neurons, where they disrupt mitochondrial function, impair synaptic integrity, and promote α-syn aggregation, thereby propagating neurodegeneration [24]. Conversely, stressed neurons also release EVs containing misfolded α-syn and damage-associated molecular patterns (DAMPs) such as ATP and heat shock proteins [26]. The pattern recognition receptors on microglia can sense these DAMPs, leading to the activation of microglia, further amplifying neuroinflammatory signaling.

Together, microglia are not merely a passive responder, but an active participant in DAergic neurodegeneration, through both soluble and vesicle-bound neurotoxic factors within the CNS and, potentially, communications with peripheral immune compartments.

### Peripheral inflammation drives microglial activation and pathology in PD

While neuroinflammation within the CNS is a hallmark of PD, accumulating clinical evidence indicates that peripheral inflammatory processes may evolve in parallel and actively influence the pathogenesis of PD [15]. Transcriptomic and immunophenotypic analyses of the peripheral blood from individuals with PD have consistently identified signatures of immune activation and imbalance, including elevated pro-inflammatory monocyte subsets, diminished natural killer (NK) cell cytotoxicity, and altered distributions of CD4^+^ and CD8^+^ T cell populations [21, 27]. Notably, increased monocytic activation and impaired NK cell function have been associated with greater motor severity, suggesting a potential link between peripheral immune status and clinical outcomes [28]. Moreover, elevated levels of circulating proinflammatory cytokines, such as interleukin (IL)-1β, IL-6, and interferon-γ (IFN-γ), have been correlated with accelerated motor and cognitive declines and may serve as early predictors of PD [28, 29].

Although peripheral immune cells may exert direct neurotoxic effects by infiltration into the CNS through impaired BBB (Fig. 1) [30] and recognition of neuronal antigens, such as major histocompatibility complex (MHC) class I-presented epitopes on DAergic neurons, these interactions cannot fully explain the complex neuroimmune pathology observed in PD. Increasing evidence suggests that microglia may play a role in translating peripheral immune signals into sustained neuroinflammation [31]. Microglia may be reprogrammed by systemic inflammatory cues to adopt a proinflammatory phenotype, thereby initiating or amplifying neuronal injury. Furthermore, neuroimaging studies have reported that in individuals with prodromal or early-stage PD, elevated peripheral levels of chemokines such as C–C motif chemokine ligand (CCL)22 and CCL26, as well as cytokines including IFN-γ, IL-1β, and IL-6, correlate with increased TSPO-PET signals indicative of microglial activation [17, 29]. These inflammatory signatures also align with markers of DAergic neurons dysfunction, as measured by dopamine transporter-SPECT or ^18^F-fluoro-L-DOPA PET [32], suggesting a close temporal and mechanistic coupling between peripheral immune dysregulation, microglial activation, and neurodegenerative processes in PD.

Collectively, these findings support a model in which peripheral immune dysregulation not only correlates with PD severity but may also act as an upstream driver of microglial activation and neuroinflammatory propagation. Notably, the interface between microglia and peripheral immune cells represents a critical axis through which systemic inflammation can influence CNS pathology. In the following section, we will summarize the cellular and molecular mechanisms underlying this bidirectional crosstalk.

### Crosstalk between microglia and peripheral immune cells

Under pathological conditions, peripheral immune cells, such as T lymphocytes, NK cells, and monocytes, can access the CNS via compromised BBB integrity or through meningeal and perivascular routes (Fig. 1) [33]. T lymphocytes, including CD4^+^ helper T cells, CD8^+^ cytotoxic T cells, and regulatory T cells (Tregs), play central roles in adaptive immunity by coordinating cellular responses, mediating cytotoxicity, and maintaining immune tolerance [30]. NK cells provide rapid, MHC-independent cytotoxicity and secrete immunomodulatory cytokines. Monocytes function as innate immune sensors and can differentiate into pro-inflammatory macrophages upon CNS infiltration [30]. Once migrating into the CNS, these peripheral immune cells establish bidirectional interactions with microglia, shaping the activation states, transcriptional signatures and effector functions of microglia, thereby influencing the neuroimmune landscape in PD [33].

#### Crosstalk between microglia and T lymphocytes

The interplay between microglia and T lymphocytes is increasingly recognized as a central axis linking innate and adaptive immune responses in PD [34]. Postmortem studies have identified infiltration of both CD4^+^ and CD8^+^ T cells into the SNc, often localized near neuromelanin-positive neurons or adjacent to blood vessels [13]. In the 1-methyl-4-phenyl-1,2,3,6-tetrahydropyridine (MPTP) mouse model, increased infiltration of CD4^+^ T cells into the SNc is accompanied by a significant loss of tyrosine hydroxylase (TH)-positive DAergic neurons, decreased striatal dopamine levels, and enhanced microglial activation marked by elevated pro-inflammatory cytokine release [35].

Among the CD4^+^ T cell subsets, the T helper 1 (Th1) cells characterized by IFN-γ secretion, promote classical M1 polarization of microglia, amplifying the production of proinflammatory mediators such as tumor necrosis factor-α (TNF-α), nitric oxide (NO), and reactive oxygen species (ROS) [15]. Furthermore, Th17 cells exacerbate neuroinflammation through IL-17 signaling and have been implicated in microglia-mediated neurotoxicity [36]. Elevated Th17 cell frequencies have been observed in both the peripheral blood and CNS of PD patients, with IL-17-expressing lymphocytes shown to induce DAergic neuronal death in patient-derived induced pluripotent stem cell models via nuclear factor kappa-light-chain-enhancer of activated B cells (NF-κB) pathway activation [37]. Notably, the microglia–CD4^+^ T cell interaction is bidirectional. Microglia can function as antigen-presenting cells via MHC class II molecules, presenting neuronal or damage-associated antigens to CD4^+^ T cells and promoting the expansion and reactivation of pro-inflammatory T cell subsets, including Th1 and Th17 cells [38]. This establishes a microglia-driven self-amplifying inflammatory loop. However, the precise mechanisms by which microglia modulate T cell function in PD remain to be fully elucidated.

Conversely, Tregs, which suppress immune activation through cytokines such as IL-10 and transforming growth factor-β (TGF-β) or through direct cell–cell contact, exert anti-inflammatory effects on microglia [30]. Clinical evidence indicates that PD patients exhibit a reduced proportion of circulating Tregs among circulating immune cells [39]. However, paradoxically, a study observed that higher circulating Treg levels in early-stage PD may correlate with more pronounced DAergic loss [39]. This may be attributed to the ability of microglia to influence T lymphocyte polarization by shaping the local cytokine milieu, thereby promoting either effector T cell phenotypes (such as CD4^+^ Th1 and Th17 cells) or Tregs depending on their activation status.

Although less extensively studied, CD8^+^ cytotoxic T lymphocytes have also been detected in the SNc of PD brains [40]. Under inflammatory conditions, neurons exhibit increased MHC class I expression, enabling direct interactions with CD8^+^ T lymphocytes. Microglia-derived cytokines such as IL-12 and IL-15 support CD8^+^ T lymphocyte activation and survival [41]. In turn, the activated CD8^+^ T cells release cytotoxic molecules including granzyme B and perforin, potentially inducing neuronal injury and influencing microglial activity via paracrine signaling [42]. In summary, these findings underscore a complex, dynamic interplay between microglia and T lymphocyte in PD, and the mutual regulation amplifies or suppresses neuroinflammation depending on the cellular context and disease stage.

#### Crosstalk between microglia and NK cells

NK cells, which are key components of the innate immune system, can infiltrate the brain parenchyma under neuroinflammatory conditions [13]. Several clinical studies have reported an increased number of circulating NK cells in PD, although their cytotoxic activity appears largely unaltered [43–45]. Postmortem analyses further identified NK cell infiltration in the SNc in PD, particularly in regions enriched with phosphorylated α-syn aggregates [13].

Experimental evidence from α-synucleinopathy mouse models has documented that NK cells are capable of internalizing extracellular α-syn, a process accompanied by elevated secretion of IFN-γ [46]. IFN-γ has been shown to act on myeloid cells including microglia, enhancing their phagocytic activity and promoting a pro-inflammatory phenotype. The activated microglia may in turn influence NK cell behavior by secreting chemokines such as CXCL10 and CCL2, which facilitate NK cell recruitment and activation within inflamed CNS compartments [47]. Furthermore, under stress or activation, microglia can express ligands such as MICA/B or RAE1 that engage NK cell activating receptors, potentially eliciting cytotoxic or immunomodulatory responses [33]. While this mechanism remains to be fully elucidated in PD, it raises the possibility of reciprocal activations between microglia and NK cells in PD-related pathology.

#### Crosstalk between microglia and monocytes

Alterations in circulating monocyte subsets and evidence of their infiltration into the brain parenchyma have been documented in PD patients [48]. However, the functional interplay between infiltrating monocytes and resident microglia remains incompletely defined. A major challenge lies in the distinction between activated microglia and infiltrating monocytes due to the overlapping surface marker profiles under inflammatory conditions [49]. Nevertheless, peripheral monocyte-enriched markers such as CCR2 and CD163 have been used for their identification in both human tissues and animal models [13, 49]. For instance, CD163^+^ monocyte-derived cells have been observed in rat models receiving 6-hydroxydopamine (6-OHDA) or α-syn injections [43]. In MPTP-induced PD models, CCR2^+^ monocytes were found to infiltrate the SNc, yet their presence alone did not exacerbate DAergic neurodegeneration. Instead, microglial CX3CR1 signaling limits the astrocyte-derived CCL2 overexpression, thereby restraining excessive monocyte recruitment and preventing monocyte-driven neurotoxicity [49]. This suggests that microglia may play a protective role by modulating the astrocyte–monocyte chemokine signaling during neuroinflammation [49].

In addition to monocytes, neutrophils, another myeloid lineage, also participate in PD-related neuroinflammation through crosstalk with microglia [15]. Recent MPTP model studies have found that elevated serum levels of TREM-1 in PD patients correlate with disease severity, and TREM-1 expression in microglia is upregulated in MPTP models [50]. Genetic deletion or pharmacological inhibition of TREM-1 attenuates DAergic neuronal loss, reduces neutrophil infiltration, and inhibits microglial activation [15]. Mechanistically, TREM-1 amplifies inflammation via the SYK–CARD9–NF-κB pathway, improving the microglia–neutrophil interactions. It may serve as a potential immunotherapeutic target in PD [50]. Therefore, elucidating the distinct yet complementary roles of microglia and monocytes in neuroimmune dynamics could offer important insights into the pathogenesis of PD.

#### Crosstalk between microglia and B cells

As key effectors of humoral immunity, B cells contribute to the neuroimmune landscape of PD by production of antibodies and cytokines [30]. In PD patients, clonal expansion of B cells, alterations in B cell subsets, and upregulation of MHC class II gene expression have been observed in both peripheral blood and cerebrospinal fluid (CSF), compared to healthy controls [30, 51]. Notably, increased titers of α-syn-reactive immunoglobulins, particularly IgG and IgM, have been reported [30]. These antibodies can traverse the compromised BBB, bind to α-syn aggregates, and engage microglial Fc gamma receptors (FcγRs), triggering the antibody-dependent cellular phagocytosis or antibody-dependent cellular cytotoxicity-like responses. Furthermore, stereotactic injection of α-syn–reactive IgG or PD patient serum into the SNc can induce microglial activation and DAergic neurodegeneration via FcγR signaling [52, 53]. In addition, autoantibodies against stress-inducible phosphoprotein 1 (STIP1)—a neuroprotective co-chaperone that also interacts with α-syn—have been detected in PD [54]. Given that STIP1 normally protects neurons against proteotoxic stress, the presence of STIP1 autoantibodies may neutralize its neuroprotective activity, thereby increasing vulnerability to α-syn-related stress and amplifying microglial activation. Beyond immunoglobulins, B cells also secrete cytokines such as IL-10, TNF-α, and lymphotoxin, which can modulate microglial activation states and the inflammatory tone [55]. Pro-inflammatory B cells may enhance the microglial pathogenic activity, whereas the IL-10–producing regulatory B cells could exert anti-inflammatory effects [55].

Although less well characterized, microglia may in turn influence B cell function. Microglia-derived cytokines, including IL-6, B cell–activating factor, and TNF-α, can regulate B cell survival, differentiation, and antibody production [30]. In addition to the BBB, these interactions may also occur in CNS border regions such as the meninges and choroid plexus—recognized immunological niches during neuroinflammatory processes. Together, these findings support a bidirectional interaction between B cells and microglia, wherein B cell-derived antibodies and cytokines modulate microglial activation, and microglial signaling shapes the humoral immune responses.

### Crosstalk between microglia and meningeal lymphatic vessels (MLVs)

The MLVs constitute a recently identified pathway for macromolecular clearance from the brain to cervical and deep cervical lymph nodes, facilitating the drainage of α-syn, immune cells, and CNS-derived antigents [56]. Idiopathic PD patients exhibit impairment of MLV-mediated clearance, associated with diminished lymph node perfusion [57]. Experimental blockade of MLVs in an α-synucleinopathy model leads to aggravated α-syn accumulation and behavioral deficits, suggesting that impaired lymphatic drainage may contribute to disease progression [58].

The role of MLVs in PD is not limited to waste clearance, but also encompasses immune surveillance and potentially shaping of the peripheral–central immune communication. Microglia appear to respond sensitively to disruptions in MLV function [21, 58]. In A53T α-syn transgenic mice, compromised lymphatic drainage is associated with pronounced microglial activation and elevated inflammatory signaling [58], indicating that impaired clearance of pathological proteins or immune mediators via MLVs may disrupt the microglial homeostasis.

Recent discoveries have underscored the intricate anatomical and functional interplay between MLVs and other CNS immune interfaces, including the meninges, choroid plexus, and skull bone marrow, which collectively coordinate immune cell trafficking and surveillance at the brain’s borders [56]. Positioned in close proximity to these interfaces, CNS-resident immune cells such as microglia and CNS-associated macrophages (CAMs) are increasingly recognized as key participants in regulating brain–periphery immune communication [59]. In particular, BAMs, a subset of CAMs residing at the meninges, perivascular spaces, and choroid plexus [60], express high levels of MHC class II and serve as principal antigen-presenting cells during α-syn-induced neuroinflammation [61]. Recent studies demonstrate that α-syn overexpression leads to BAM expansion and activation. Depletion experiments showed that selective depletion of BAMs attenuates CD4^+^ T cell recruitment and downstream inflammatory responses [61]. These findings highlight that BAMs link brain-derived antigens to peripheral lymphatic drainage and adaptive immune activation, implicating a functional coupling between BAMs and the MLV network.

Meanwhile, microglia, embedded within the CNS parenchyma, are well positioned to integrate signals from both local neuronal stress and peripherally derived immune or lymphatic cues [21]. Although the role of the MLV system in neuroimmune communication in PD remains underexplored, it is plausible that impaired lymphatic drainage could modulate the microglial activation state, while reactive microglia, in turn, might influence the meningeal or vascular environments (Fig. 1). Future studies using spatial transcriptomics, in vivo imaging, and targeted manipulation of lymphatic structures will be essential to delineate the coordinated roles of microglia, BAMs, and MLVs in the initiation and propagation of neuroinflammation [62].

## Role of microglia in the α-syn “brain-first/body‐first” hypothesis

**Fig. 2:**
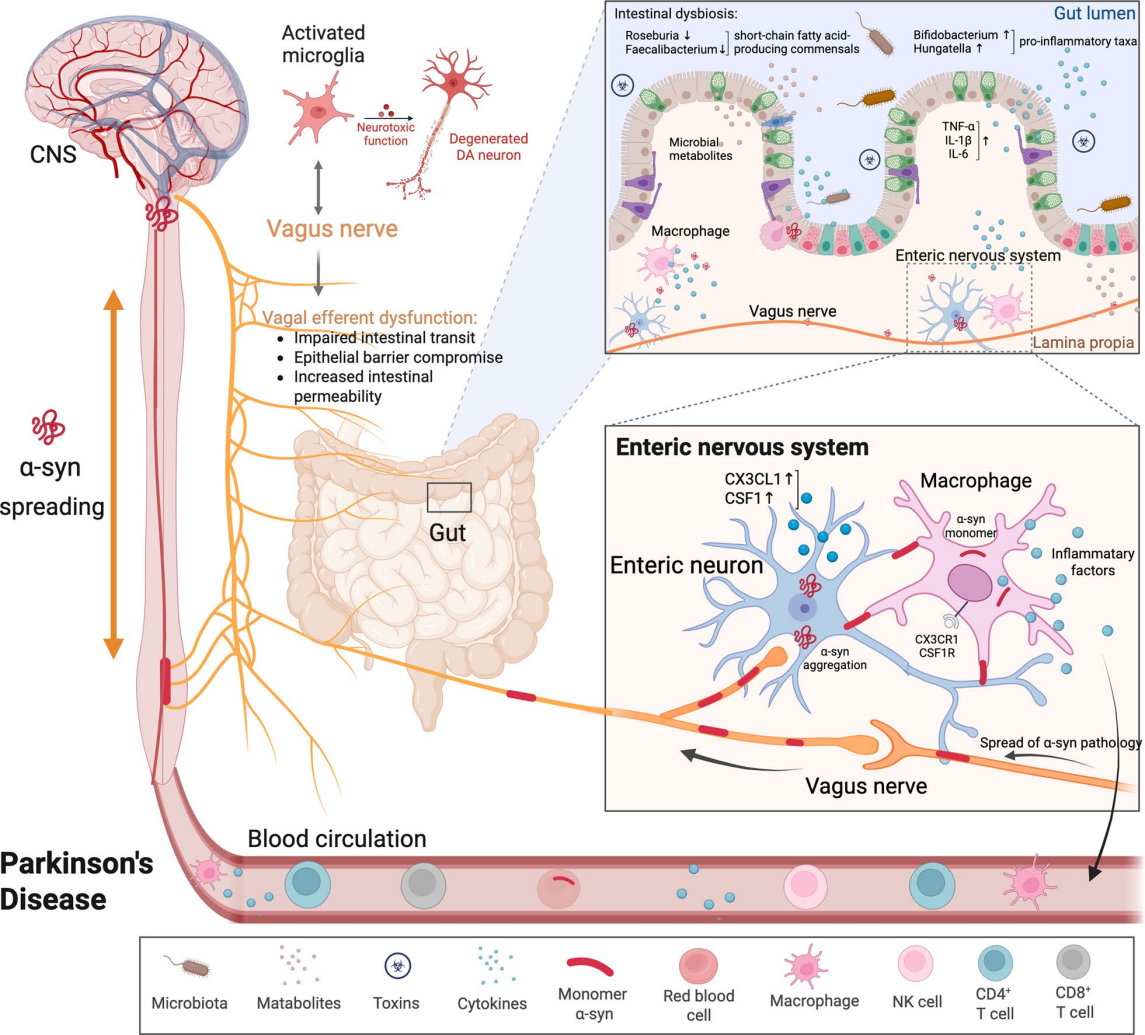
Microglia as a central mediator of α-syn propagation along the gut–brain axis. Intestinal dysbiosis in PD patients is characterized by depletion of short-chain fatty acid-producing commensals such as *Roseburia* and *Faecalibacterium*, and enrichment of pro-inflammatory taxa such as *Bifidobacterium* and *Hungatella*, which correlates with intestinal inflammation. In addition, pathological α-syn originating in the gut can accumulate within enteric neurons and initiate local immune activation. Gut-resident macrophages respond to α-syn pathology and shape the local immune milieu, influencing whether α-syn aggregates are contained or transmitted toward the CNS via the vagus nerve. Sustained exposure to pathological α-syn and chronic peripheral immune signals, including microbial metabolites and circulating cytokines, drives microglia toward a proinflammatory state and facilitates prion-like spread of α-syn. In turn, dysfunctional microglia exacerbate gut barrier dysfunction through vagal efferent and disrupt microbial homeostasis, establishing a vicious cycle of gut–brain neuroimmune dysregulation. Figure created with BioRender.com

The “brain-first” and “body-first” hypotheses of PD propose two distinct trajectories of α-syn pathology propagation: one originating in the CNS, particularly the olfactory bulb or amygdala, and the other starting in peripheral tissues such as the gastrointestinal tract, with subsequent spread to the brain via autonomic pathways such as the vagus nerve [3, 63]. Accumulating research has highlighted microglia as an active participant in transmitting α-syn neuropathology between cells [6, 64]. Through a repertoire of pattern recognition receptors, notably Toll-like and NOD-like receptors, microglia detect and internalize extracellular α-syn [65, 66], and mount innate immune responses that can influence the clearance or spread of pathogenic species (Fig. 2). In this context, microglia may shape the microenvironmental permissiveness for α-syn propagation in a subtype-specific and regionally distinct manner.

### Microglia in the “brain-first” hypothesis

In “brain-first” subtype of PD, early involvement of limbic and cortical regions with relatively high microglial density and transcriptional heterogeneity suggests that the microglial state may contribute to region-specific vulnerability [67]. Single-cell RNA sequencing studies have revealed substantial microglial diversity across brain regions in PD mouse model, with distinct inflammatory, metabolic, and phagocytic profiles [6, 68]. In particular, microglia in vulnerable regions, such as the SNc, tend to adopt pro-inflammatory states that not only exacerbate local neurotoxicity but also facilitate the propagation of pathological α-syn [69].

Activated microglia release pro-inflammatory cytokines such as IL-1β, TNF-α, and IL-6, along with ROS and NO, which can promote α-syn aggregation through oxidative and nitrative post-translational modifications [66]. Moreover, microglia contribute to the intercellular spread of α-syn via secretion of exosomes and the formation of tunneling nanotubes [70]. Microglia also express lymphocyte activation gene 3 (LAG3), a receptor that preferentially binds to aggregated α-syn [71, 72]. Blockade of LAG3 with monoclonal antibodies attenuates neurodegeneration, suggesting that the microglial LAG3 is a mediator of α-syn uptake and propagation between cells [73]. Furthermore, recent structural and functional studies have identified the receptor for advanced glycation end products (RAGE) as a key mediator of microglial inflammatory responses to α-syn fibrils. Interactions between the V domain of RAGE and the acidic C-terminus of α-syn triggers neuroinflammation, and this effect is attenuated by RAGE inhibition [74].

At the molecular level, misfolded α-syn species act as DAMPs that are recognized by pattern recognition receptors such as Toll-like receptor 2 (TLR2) on microglia [65]. Engagement of TLR2 activates the NF-κB signaling cascade, inducing transcription of inflammasome components including nucleotide-binding domain-like receptor protein 3 (NLRP3), poptosis-associated speck-like protein (ASC), and pro-caspase-1 [75]. Assembly of the NLRP3 inflammasome subsequently activates caspase-1, promoting the maturation and secretion of IL-1β [23]. In addition, transcriptomic analysis has confirmed upregulation of key genes associated with both the NF-κB pathway and the inflammasome complex in CX3CR1-SNCA transgenic mice, which express human α-syn selectively in microglia [76]. These findings indicate that microglia serve as sensors of extracellular α-syn, amplifying pathogenic effects through intrinsic inflammatory signaling.

Notably, recent evidence suggests that microglia may also contribute to the peripheral dissemination of α-syn pathology in the brain-first subtype [77]. In a model of brain-initiated α-synucleinopathy, α-syn aggregates are detected in the ileum, localized within CD11c^+^ immune cells [77]. Single-cell transcriptomic analysis revealed that these ileal CD11c^+^ cells share transcriptional profiles with activated microglia, including enrichment of inflammatory and antigen presentation signatures [77]. Using Dendra2 photoconversion-based lineage tracing, researchers demonstrated that a subset of CD11c^+^ cells migrated from the brain to the gut, providing direct evidence for CNS-to-periphery cellular trafficking [77]. These results suggest that microglia-like cells contribute not only to central neuroinflammation and α-syn propagation, but also to peripheral immune priming and systemic disease extension. Taken together, these findings underscore the multifaceted role of microglia in the “brain-first” subtype of PD. Microglia not only serve as local effectors of neuroinflammation, facilitating α-syn aggregation and propagation within the brain, but also mediate CNS-to-peripheral immune communication.

### Microglia in the “body‐first” hypothesis

In the “body-first” subtype of PD, α-syn pathology is hypothesized to originate in the ENS, from which it propagates centrally via transsynaptic transmission along the vagus nerve [4]. Supporting this model, studies in rodents have demonstrated that truncal vagotomy can block the transmission of pathological α-syn to the brain and attenuate the development of motor deficits [78]. Furthermore, vagus nerve stimulation exerts neuroprotective effects in PD models, including preservation of TH-positive DAergic neurons and amelioration of motor deficits [79]. These effects are, at least in part, attributed to the modulation of neuroinflammatory processes, as evidenced by reduced microglial activation following vagus nerve stimulation [80].

Within the ENS, gut injection of α-syn not only induces phosphorylated α-syn accumulation in enteric neurons, but also elicits local immune responses [81]. Specifically, it upregulates CX3CL1 and CSF1, which interact with CX3CR1 and colony-stimulating factor 1 receptor (CSF1R) expressed on ENS-resident macrophages [81]. These macrophages, functionally analogous to microglia in the CNS, are closely associated with enteric neurons, and exhibit enriched expression of genes involved in vesicular trafficking and lysosomal function, including PD risk genes *GBA1* and *LRRK2* [82]. The gut-resident macrophages may actively shape the local immune response to α-syn and influence the subsequent propagation of α-syn toward the CNS. However, evidence directly linking these processes to α-syn propagation remains limited. The shared transcriptional programs, sensitivity to α-syn, and engagement of common innate immune pathways between ENS-resident immune cells and CNS microglia point to the existence of a coordinated neuroimmune axis bridging the gut and brain [83]. However, the underlying mechanisms governing this immunological crosstalk remain poorly understood and warrant further investigation.

Upon entry into the CNS, α-syn aggregates encounter microglia, particularly in the brainstem during the body-first disease trajectory. The microglia then exhibit region-specific transcriptional and functional profiles that shape their immune responses to the pathogenic protein [84]. Similar to the brain-first subtype, these microglial responses may play a dual role in either containing or facilitating the spread of α-syn pathology. Initially, microglia may attempt to restrict pathology through phagocytosis and degradation of α-syn aggregates. However, chronic activation of microglia may shift them toward a neurotoxic phenotype characterized by sustained secretion of proinflammatory cytokines, impaired phagocytic function, and facilitation of prion-like spread [64].

Furthermore, emerging evidence highlights red blood cells (RBCs) as a peripheral source of α-syn, harboring levels far exceeding that present in the CSF [85, 86]. Systemic exposure to lipopolysaccharide (LPS) enhances the transport of RBC-derived EVs into the brain via adsorptive transcytosis. Within the CNS, these vesicles localize to microglial populations and elicit marked inflammatory responses [7]. Furthermore, EVs isolated from the peripheral blood of PD patients provoke a more robust activation of microglia than those from healthy controls, suggesting that peripheral α-syn may act as a pathological cue that primes or exacerbates microglia-mediate neuroinflammation and contribute to disease progression [85].

Collectively, these observations position microglia as a central regulator of α-syn pathobiology. Through their regional-specific transcriptional states and bidirectional interactions with the peripheral immune network, microglia may not only shape the neuroinflammatory environment, but also influence the site of α-syn initiation and the directionality of its propagation. However, the precise mechanisms by which microglia determine a “brain-first” or “body-first” trajectory in individual patients remain incompletely understood. Future studies using spatial transcriptomics, longitudinal neuroimaging, and cell-type-specific functional manipulations across disease stages are essential to elucidate the role of microglia in PD subtype divergence and progression.

## Microglia play a role in the microbiota-gut-brain interactions in PD

The bidirectional communications between the gut and brain during the progression of PD are increasingly recognized to be modulated by microbial metabolite activity and gut microbial homeostasis (Fig. 2)  [87]. Intestinal dysbiosis is consistently found in PD patients, characterized by depletion of short-chain fatty acid (SCFA)-producing commensals such as *Roseburia* and *Faecalibacterium*, and enrichment of pro-inflammatory taxa such as *Bifidobacterium* and *Hungatella*, which correlate with intestinal inflammation and disease severity [88, 89]. Similarly, experimental PD models using neurotoxins such as MPTP or rotenone exhibit microbiota alterations, supporting a relationship between gut microbial imbalance and PD-related neuropathology [90, 91].

Accumulating evidence indicates that gut microbiota can influence CNS function via multiple mechanisms, including neural signaling, endocrine regulation, and modulation of systemic and neuroinflammatory responses [92]. Microglia are particularly responsive to gut-derived microbial metabolites and peripheral immune signals, which critically shape their ontogeny, activation, and immunomodulatory function [93]. Conversely, microglial reactivity can impact the gut barrier integrity and microbiota composition (Fig. 2).

### Microglia mediate the role of gut microbiota in PD

Gut microbiota and their metabolites play critical roles in regulating microglial development and function [92]. Germ-free (GF) mice, which lack intestinal microbiota, exhibit widespread microglial abnormalities, including altered proportions of microglial subpopulations, immature phenotypes, and impaired innate immune responses [94]. This underscores the essential role of microbial signals in shaping microglial homeostasis. In addition, GF mice display decreased expression of genes related to immune function and a lack of an age effect on microglial morphology and oxidative stress [95]. These findings suggest that microbiota-derived signals are required for microglial activation and age-related adaptation.

In PD, there is growing evidence supporting the notion that the gut microbiota–microglia axis contributes to disease pathogenesis [92]. For example, *Blautia producta*, a butyrate-producing commensal bacterium, attenuates the microglia-mediated neuroinflammation in PD models by modulating the RAS–NF-κB signaling pathway [96]. Besides, fecal microbiota transplantation (FMT), while initially developed as a therapy for gastrointestinal disorders, has emerged as a candidate intervention for PD and has been evaluated in several randomized controlled trials for its impact on motor and non-motor symptoms [97, 98]. Furthermore, transplantation of fecal microbiota from PD patients into α-syn-overexpressing A53T mice led to increased gut permeability, enhanced intestinal inflammation and neuroinflammation, and microglial activation, accompanied by DAergic neuron loss. These effects were associated with activation of the TLR4/NF-κB/NLRP3 pathway in both the colon and the brain [99]. Similarly, in a chronic rotenone PD model, FMT treatment restored gut microbial composition, alleviated gastrointestinal symptoms, and improved motor function. Notably, FMT reduced systemic inflammation, protected the integrity of the BBB, and suppressed microglial activation in the SNc, partially via inhibition of the LPS–TLR4/MyD88/NF-κB signaling axis [100]. In conclusion, these findings demonstrate that FMT can ameliorate PD-related pathology by modulating peripheral inflammation and central microglial responses, reinforcing the therapeutic relevance of the gut–microglia axis in PD.

While it is evident that the gut microbiota shape microglial phenotypes and inflammatory states, the influence of microbiota on PD likely extends beyond immunomodulation that remains undiscovered. Emerging studies suggest that microbial metabolites may also impact other key neurodegenerative processes, including autophagy [101], proteasome function [102] and protein aggregation pathways closely linked to PD pathogenesis. Deciphering how gut microbiota influence microglial activation in conjunction with cellular proteostasis, neurotransmitter metabolism, and immune signaling may provide a more integrated understanding of PD as a multifactorial neurodegenerative disorder.

### The influence of microglia on the microbiota in PD

While the gut microbiota exert a well-established effect on microglial maturation and activation, emerging evidence indicates that microglia may also exert effects on gut microbial composition, particularly under neuroinflammatory conditions such as in PD [103]. Microglial activation can disrupt autonomic homeostasis via altered hypothalamic and brainstem signaling, leading to downstream dysfunction in gastrointestinal motility, secretion, and barrier integrity—key factors that govern the structure and resilience of the gut microbiota [87, 104]. This effect is partly mediated by the vagus nerve, a major bidirectional conduit between the CNS and the gastrointestinal tract [105]. A cholinergic anti-inflammatory pathway through the vagus nerve has been identified. This pathway reduces peripheral inflammation and intestinal permeability, potentially altering the composition of the microbiota [106].

In PD models, heightened microglial reactivity has been associated with vagal efferent dysfunction, resulting in impaired intestinal transit, epithelial barrier compromise, and increased intestinal permeability (Fig. 2) [104]. These create an environment conducive to microbial dysbiosis, characterized by the loss of commensal bacteria and overgrowth of pro-inflammatory taxa. Furthermore, microglia-derived inflammatory mediators such as IL-1β and TNF-α may influence the brain–gut axis via vagal afferents, altering the central autonomic circuits that regulate gut function. Notably, vagotomy in animal models attenuates gut inflammation and microbial shifts induced by CNS pathology [107], highlighting the importance of the vagus nerve in microglial modulation of gut microbiota. Conversely, vagal stimulation has demonstrated anti-inflammatory effects on both the gut and the brain, potentially by inhibiting microglial activation [80]. These findings suggest that microglia, through interactions with the autonomic nervous system, play an active role in shaping the gut microbial ecology in PD, contributing to neuroinflammation and gut dysbiosis.

In summary, microglia serve as a central effector in the bidirectional communication between the gut microbiota and the brain (Fig. 2). Microglial responses to gut-derived inflammatory or metabolic cues may drive neuroinflammatory cascades, exacerbate α-syn pathology, and contribute to BBB breakdown.

## Microglia in multi-factorial crosstalk networks in PD: a double-edged sword

As discussed above, microglial and systemic inflammation, α-syn aggregation and propagation, and gut-derived immune and metabolic alterations are key drivers of PD pathogenesis. However, they do not act in isolation. Converging evidence suggests that microglia integrate signals from multiple pathological domains, thereby functioning as a central mediator [10]. In addition, it is increasingly recognized that microglia also play neuroprotective roles, contributing to tissue repair and immune homeostasis, depending on the spatiotemporal context and nature of the stimuli. The phenotypic and functional heterogeneity of microglia makes them a critical node within the broader neuroimmune landscape of PD.

### Microglial states, phenotypic plasticity and neuroprotective roles

The classical M1/M2 dichotomy of microglia does not adequately capture the complexity of microglial activation in vivo. Recent single-cell transcriptomic studies have identified a spectrum of disease-associated microglial (DAM)-like states, initially described in AD, and increasingly recognized in PD. These states are characterized by transcriptional profiles related to phagocytosis, lipid metabolism, and immune regulation [108]. However, their precise roles in α-syn clearance and PD progression remain incompletely defined.

Microglia display remarkable phenotypic plasticity, transitioning between pro-inflammatory and neuroprotective states depending on the microenvironmental cues. Under anti-inflammatory signals such as IL-10, TGF-β, and neuron-derived CX3CL1, microglia express increased levels of neurotrophic factors including brain-derived neurotrophic factor (BDNF), glial cell-derived neurotrophic factor, and insulin-like growth factor-1, supporting DAergic neuron survival. For example, studies have shown that pharmacological activation of CX3CR1 signaling attenuates microglia-mediated neuroinflammation and protects against neurodegeneration in PD models [109, 110]. Additionally, microglia play a key role in clearing extracellular α-syn aggregates through phagocytosis and autophagy pathways. Notably, failure in this clearance capacity has been linked to α-syn propagation and exacerbation of neurodegeneration. Furthermore, emerging evidence highlights that, in addition to carrying pathogenic molecules, EVs derived from microglia can also deliver protective factors, including TGF-β, BDNF, and regulatory microRNAs. For example, EVs enriched with miR-100‑5p, derived from adipose-derived stem cells in a PD mouse model, suppress microglial activation and attenuate DAergic neurodegeneration by targeting the DTX3L/STAT1 pathway [111]. Similarly, EVs containing miR-106b improve neuronal autophagy and survival via downregulation of CDKN2B in PD models [112].

Microglia exhibit dynamic and region-specific functional states in PD, with those in the SNc displaying stronger pro-inflammatory and phagocytic profiles compared to those in other brain regions. This is likely due to the differences in α-syn burden, neuronal susceptibility, and peripheral immune signals [19]. Deciphering the mechanisms underlying these phenotypic transitions may offer novel therapeutic strategies to modulate neuroinflammation and preserve DAergic neuron integrity in PD.

### Microglia in the multi-factorial crosstalk networks: integration of central and peripheral signals

Microglia function as key transducers of peripheral–central immune crosstalk in PD, dynamically integrating pathological signals including α-syn aggregates, systemic cytokines, and gut microbial metabolites. These inputs converge on innate immune pathways—such as TLRs, NF-κB, STING, and inflammasomes, which collectively shape microglial activation states and phenotypic plasticity [19]. This multifactorial signaling network plays a pivotal role in modulating PD pathogenesis.

A seminal study by Sampson et al. [113] reported that GF α-syn-overexpressing mice exhibit attenuated microglial activation, reduced α-syn aggregation, and improved motor performance compared to mice with an intact microbiome. Restoration of SCFAs in GF mice promotes microglial activation, α-syn pathology, and motor deficits. This indicates that microbial metabolites drive microglia toward a pro-inflammatory, reactive phenotype that promotes α-syn propagation and neurodegeneration. Conversely, microbiota depletion inhibits the microglia-mediated neuroinflammation. Notably, FMT from PD patients further induced microglial activation toward pathogenic phenotypes, characterized by elevated inflammatory gene expression [113], supporting the notion that the gut-derived metabolites critically modulate microglial phenotypes, promoting maladaptive neuroinflammation and α-syn propagation.

A subsequent study has highlighted the impact of gut-derived metabolic dysfunction on microglial states in a gut-originated α-syn aggregate PD murine model [114]. Overgrowth of *Dubosiella newyorkensis* disrupted branched-chain amino acid (BCAA) metabolism, leading to systemic BCAA accumulation. This metabolic stress induced mTOR hyperactivation in microglia, impairing lysosomal function—a critical pathway for α-syn clearance. As a result, microglia shifted toward a dysfunctional phenotype with impaired phagocytosis, preventing effective degradation of α-syn aggregates, thereby accelerating disease progression. Microbiota depletion by antibiotics restored microglial lysosomal function and reduced their pro-inflammatory state, underscoring the microglial phenotypic plasticity under metabolic regulation [114].

Overall, these findings outline a complex crosstalk network in which α-syn pathology, gut microbiota, peripheral metabolic dysregulation, and systemic immune signals converge to shape the microglial activation states in PD. Microglia, in turn, act as both sensors and effectors within this network, translating peripheral cues into central neuroimmune responses that can either exacerbate or mitigate α-syn pathology and neurodegeneration. Understanding how peripherally derived factors dynamically influence microglial phenotype may uncover novel therapeutic strategies targeting the microbiota–immune–brain axis in PD.

## Therapeutic implications and conclusion

The recognition of microglia as a central integrator in the bidirectional communication between the peripheral immune system and the CNS highlights their potential as a therapeutic target for PD. However, the functional plasticity of microglia depending on the microenvironment poses significant challenges for therapeutic targeting. Therapeutic strategies that suppress microglial activation, such as NLRP3 inflammasome inhibitors, glucagon-like peptide-1 receptor agonists, myeloperoxidase inhibitors, and C–C chemokine receptor type 3 antagonists, have demonstrated efficacy in preclinical PD models (Table 1) [115, 116]. Several of these agents have progressed to early-phase clinical trials, showing acceptable safety profiles and, in some cases, encouraging signs of therapeutic benefit (Table 1). Nonetheless, inhibition of microglial activation has inherent risks, including impairment of essential homeostatic functions such as debris clearance, synaptic remodeling, and trophic support [10]. Thus, promising strategies may involve reprogramming microglia toward or maintaining them in a homeostatic, neuroprotective state.

**Table 1:** Clinical trials and preclinical studies targeting central and peripheral immune systems in PD

Category/target	Intervention/drug	Study type (models/patients)	Key findings	Mechanism	Trial ID/references
NLRP3 inflammasome inhibition	Dapansutrile	Phase II, PD patients	Ongoing trial with PET/CSF endpoints	NLRP3 modulation in microglia and reduced inflammation in the CSF and blood	NCT07157735
	Selnoflast (RO7486967)	Phase Ib, early PD (ongoing)	Ongoing trial with PET/CSF/DaT endpoints		ISRCTN85338453 NCT05924243
	NT-0796 (NodThera)	Phase Ib/IIa, PD patients	Reduced inflammatory markers in blood and CSF after 28 days; well tolerated		Company press release 2024–03–07
GLP-1R modulation	NLY01 (GLP-1R agonist)	Phase II, early drug-naïve PD	No significant effect on primary endpoints; a potential treatment effect was observed in a subgroup of younger patients	Targeting the microglial activation cascade	NCT04154072 [115]
MPO inhibitor	AZD3241 (MPO inhibitor)	Phase II, PD patients	Reduced microglial activation evaluated by PET	Suppression of microglial activity and improvement of dopaminergic cell survival	NCT01603069
Antagonist of CCR3	AKST4290	Phase II, PD patients	Failed to meet the primary endpoint of clinical efficacy	Reducing T cell infiltration into the brain and decreasing microgliosis	NCT04369430
Peripheral immune modulation (GM-CSF)	Sargramostim	Phase Ia/Ib, PD patients	Increased Tregs, improved immune profile; clinical stabilization signals	Peripheral immune modulation and microglial reprogramming	NCT03790670; NCT05677633
Inhibiting lymphocyte proliferation	Azathioprine	Phase II, PD patients	Completed in February 2024, but results are not yet available	Broadly inhibits the proliferation of T and B lymphocytes	ISRCTN14616801
Regulation of ERK	NE3107	Phase II, PD patients	An improvement of MDS-UPDRS-III score was noted in 80% of the treatment arm compared to 63.6% of the placebo arm	Regulation of ERK-mediated inflammatory pathways	NCT05083260
Peripheral immune modulation (VIPR2)	LBT-3627 (VIPR2 agonist)	Phase Ia/Ib PD trials	Ongoing trial with PET/CSF/DaT endpoints	Peripheral immune biasing alters the central microglial phenotype	NCT06466525
Soluble TNF inhibition	XPro1595 (DN-TNF)	Preclinical study, monkey and 6-OHDA rat models	Reduced neuroinflammation and DA neuron loss in models	Peripheral TNF inhibition and influencing central microglia	[117, 119]
Broad-spectrum antibiotic	Minocycline	Preclinical studies	Reduced microglial activation and neuroinflammation in PD models	Insufficient for PD modification	[118]
Gut microbiota intervention	FMT	Multicenter RCT in early PD	FMT was safe but did not offer clinically meaningful improvements	Modulation of the gut-immune-microglia axis in patients	NCT03808389 [97]
Gut microbiota intervention	Rifaximin	Phase II, PD patients	Ongoing trial	Antibiotic modulation of gut flora impacts peripheral inflammation and microglia	NCT03575195

GLP-1R, glucagon-like peptide-1 receptor; MPO, myeloperoxidase; CCR3, C–C chemokine receptor type 3; GM-CSF, modulation of granulocyte macrophage–colony stimulating factor; VIPR2, vasoactive intestinal peptide receptor 2; ERK, extracellular signal-regulated kinase; 6-OHDA, 6-hydroxydopamine; FMT, fecal microbiota transplantation; RCT, randomized controlled trial

Accumulating preclinical and clinical evidence also supports the feasibility of peripheral immune modulation as an indirect means to reshape microglial phenotypes [117–119]. For example, modulation of GM-CSF (granulocyte macrophage–colony stimulating factor), VIPR2 (vasoactive intestinal peptide receptor 2), or ERK (extracellular signal-regulated kinase) signaling pathways promotes an anti-inflammatory microglial phenotype, and has shown potential benefits in early clinical studies (Table 1) [116]. Similarly, gut microbiota-targeting interventions, such as microbial modulation or FMT, have been evaluated in both animal models and pilot human studies [97]. Furthermore, preclinical studies have identified novel therapeutic strategies. A recent study demonstrated that prophylactic immunization with wild-type α-syn promotes the expansion of dopamine receptor D3^+^ Tregs in the periphery, accompanied by enhanced STAT5 signaling. This immunomodulatory shift is associated with a transition of brain microglia toward a tolerogenic phenotype (CD200R⁻CD54^+^CD103^+^CD172a^+^), resulting in attenuation of neuroinflammation and preservation of DAergic neurons [120]. This finding underscores the therapeutic potential of harnessing the interactions between peripheral T cells and microglia for CNS immune regulation and neuroprotection [120].

In parallel, bioengineered nanotherapeutics are being developed to simultaneously modulate central and peripheral immune functions [24]. A notable example is the EV-based nanoformulation (EVN) strategy, comprising CCR2-enriched mesenchymal stem cell-derived EVs conjugated to a nanocarrier loaded with dihydrotanshinone I. This dual-functional platform exhibits targeted accumulation in the SNc via CCL2–CCR2 interactions, thereby limiting peripheral monocyte infiltration. Simultaneously, activation of the Nrf2–GPX4 pathway within microglia mitigates ferroptosis-driven neuroinflammation [121]. In PD mouse models, EVN treatment effectively reduced microglial-mediated inflammation, preserved DAergic neuron integrity, and improved motor performance. The EVN represents a systems-level therapeutic approach that integrates peripheral immune modulation with direct neuroprotection [121].

## Conclusion

Microglia dynamically integrate signals from misfolded α-syn, systemic immune signals, and gut-derived metabolites, and shape neuroimmune responses that critically influence disease progression of PD. This underscores the necessity of therapeutic strategies that simultaneously target both central microglial dysfunction and peripheral pathological drivers. Future efforts should prioritize precision approaches that consider the spatial, temporal, and molecular heterogeneity of microglial states within the broader context of the microbiota–immune–brain axis. Interventions aimed at recalibrating microglial phenotypic states, whether via modulation of peripheral immunity, correction of microbial or metabolic imbalances, or advanced delivery platforms, hold considerable promise for achieving meaningful disease modification.

## Data Availability

Not applicable.

## Abbreviations

BAMs: Border-associated macrophages
BBB: Blood‐brain barrier
BCAA: Branched-chain amino acid
BDNF: Brain-derived neurotrophic factor
CAMs: CNS-associated macrophages
CCL: C–C motif chemokine ligand
CSF: Cerebrospinal fluid
CSF1R: Colony-stimulating factor 1 receptor
CNS: Central nervous system
CX3CL1: Chemokine (C-X3-C motif) ligand 1
DAergic: Dapaminergic
ENS: Enteric nervous system
EVN: Extracellular vesicle-based nanoformulation
DAM: Disease-associated microglial
DAMPs: Damage-associated molecular patterns
ERK: Extracellular signal-regulated kinase
EVs: Extracellular vesicles
FcγRs: Fc gamma receptors
FMT: Fecal microbiota transplantation
GF: Germ-free
IFN-γ: Interferon -γ
IL: Interleukin
LAG3: Lymphocyte activation gene 3
LPS: Lipopolysaccharide
MHC: Major histocompatibility complex
MLVs: Meningeal lymphatic vessels
NLRP3: Nucleotide-binding domain- like receptor protein 3
NK: Natural killer
NO: Nitric oxide
NF-κB: Nuclear Factor kappa-light-chain-enhancer of activated B cells
Nurr1: Nuclear receptor related 1
PD: Parkinson’s disease
PET: Positron emission tomography
PNS: Peripheral nervous system
RAGE: Receptor for advanced glycation end products
ROS: Reactive oxygen species
SCFA: Short-chain fatty acid
SNc: Substantia nigra pars compacta
STIP1: Stress-inducible phosphoprotein 1
TH: Tyrosine hydroxylase
Th1: T helper 1
TGF-β: Transforming growth factor-β
TLR: Toll-like receptor
Tregs: Regulatory T cells
TNF-α: Tumor necrosis factor-α
MPTP: 1-Methyl-4-phenyl-1,2,3,6-tetrahydropyridine
6-OHDA: 6-Hydroxydopamine
